# Transient asystole during balloon dilation of the Eustachian tube: A case report

**DOI:** 10.1097/MD.0000000000031720

**Published:** 2022-11-04

**Authors:** Mee Young Chung, Min Jung Shin, Seung Hee Cha, Ji Yung Lee

**Affiliations:** Department of Anesthesiology and Pain Medicine, College of Medicine, The Catholic University of Korea, Seoul, Korea.

**Keywords:** asystole, balloon dilation, Eustachian tube, general anesthesia, reflex, vagal

## Abstract

Neurally mediated reflexes can induce cardiac arrest during head and neck surgery through mechanisms including direct vagal stimulation, trigeminocardiac reflex, and baroreceptor reflex. Balloon dilation of the Eustachian tube (BDET) is a safe procedure without serious complications, including cardiac arrest.

**Patient concerns:**

Transient asystole developed during BDET under general anesthesia in a 33-year-old woman as soon as the balloon in the Eustachian tube (ET) was inflated.

**Diagnoses:**

Monitoring records were reviewed. The asystolic period was recorded on the patient monitor as an event, which we recalled and printed. The asystole lasted for 13 seconds.

**Interventions:**

The patient recovered sinus rhythm spontaneously after the balloon was deflated before resuscitation. The BDET was successfully performed after prophylaxis with vagolytic drugs.

**Outcomes:**

The patient recovered uneventfully after anesthesia.

**Lessons:**

BDET, previously known to be a relatively safe procedure, induces asystole via balloon dilation. It is thought to be a neurally mediated vagal reflex, and both anesthesiologists and otologic physicians should pay proper attention to monitoring during the procedure.

## 1. Introduction

The incidence of intraoperative cardiac arrest in non-cardiac surgeries, of which 20% are otolaryngologic surgeries, was estimated to be 7 per 10,000 surgeries. The proposed mechanisms of intraoperative cardiac arrest in head and neck procedures include direct vagal stimulation, trigeminocardiac reflex, and baroreceptor reflex.^[1]^ Balloon dilation of the Eustachian tube (BDET) has been demonstrated to be efficacious in patients with Eustachian tube dysfunction (ETD).^[2]^ It has a low major complication rate of 0.3% due to balloon failure, and a minor complication rate of 1.7%.^[3]^ As a new procedure with a good safety profile, it is proposed to be performed in an office setting under local anesthesia.^[4]^ However, we recently experienced a patient with transient asystole during BDET, an event that alarms anesthesiologists. This case report describes our experience with transient asystoles during BDET and a literature review.

## 2. Case presentation

**Figure 1.**
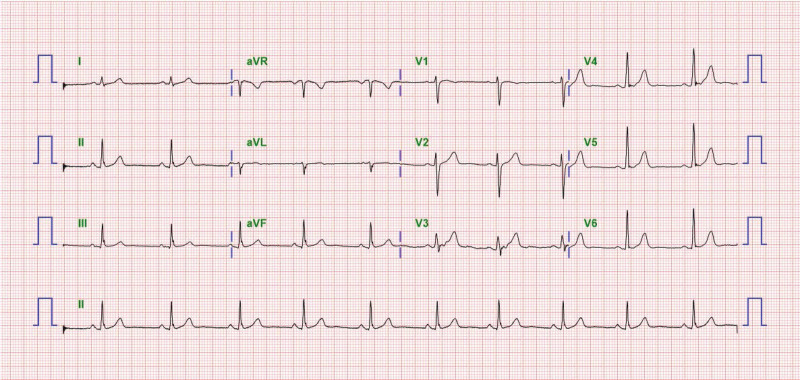
Preoperative EKG shows normal EKG. EKG = electrocardiogram.

A 33-year-old woman was scheduled to undergo left tympanoplasty and left BDET. Preoperative evaluation, including chest radiography and all laboratory values, was normal. Her electrocardiogram (EKG) results were normal (Fig. 1). She had undergone v-tube insertion in the left ear 27 years earlier.

Upon arrival at the operating room, her vital signs were as follows: blood pressure (BP) 113/72 mm Hg; heart rate (HR) 79 beats/minute, and oxygen saturation, 99%. Remifentanil was infused at a rate of 0.5 microgram/kg/minute during preoxygenation. After 120 mg of propofol injection followed by a decrease in the bispectral index and a 50-mg injection of rocuronium bromide, the trachea was intubated with an armored tube with an internal diameter of 7.0 mm. Her vital signs were stable at BP 112/79 mm Hg, HR 69 beats/minute, oxygen saturation 99%, and regular sinus rhythm on the EKG after anesthesia induction. The anesthetic drugs were 6 vol% desflurane in an O_2_-air mixture at an FiO_2_ of 40% with remifentanil supplementation infused at a rate of 0.1 to 0.15 microgram/kg/minute. Fifteen minutes later, the left BDET was started under nasal endoscopic view. At that time, her vital signs were BP 91/48 mm Hg, HR 70 beats/minute, and oxygen saturation 100%. The surgeon inserted the balloon into the left ET and inflated it until the intraballoon pressure increased to 12 atm. As soon as the target pressure was reached, we observed a sudden onset of asystole on the patient monitor. Because there was no radial pulse, we confirmed which procedure the surgeon performed and asked the surgeon to deflate the balloon immediately, remove all devices from the operative field, and prepare chest compressions. Fortunately, the heart started to beat spontaneously before the chest compressions and then progressively recovered to sinus rhythm. Because the left BDET was an essential procedure for the patient, the surgeon asked if the procedure could be repeated. Considering the possibility of a vagally mediated reflex, we injected glycopyrrolate 0.2 mg. Her vital signs were BP 124/79 mm Hg, HR 37 beats/minute, and oxygen saturation 100%. We injected 0.25 mg of atropine to increase heart rate. In addition, a transcutaneous pacemaker was prepared to treat asystole if necessary. After the HR increased to 60 beats/minute, the left BDET was restarted. At that time, there were no changes in the HR. Subsequent tympanoplasty was uneventful. After discontinuation of all anesthetics and injection of 100 mg of sugammadex, the patient recovered from anesthesia and was extubated. During recovery, we asked her about the history of her syncope and reported that she had experienced syncope about 10 years earlier, which was not evaluated further. She thought she was very healthy and refused further cardiologic and neurological diagnoses.

**Figure 2.**
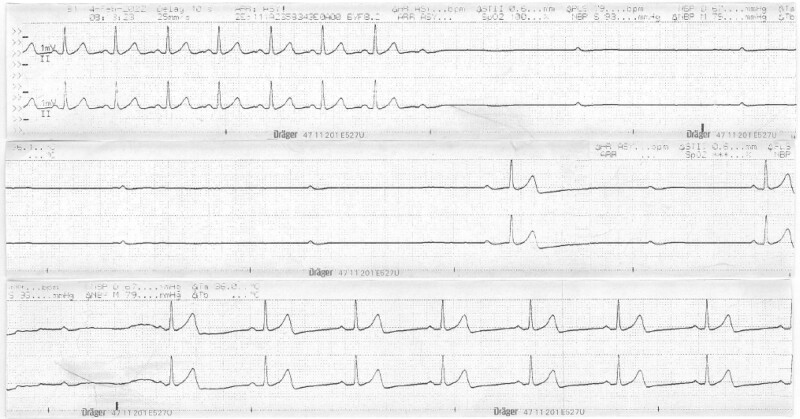
The printed EKG shows transient asystole for 13 seconds and the spontaneous recovery of sinus rhythm. EKG = electrocardiogram.

Monitoring records were reviewed. The asystolic period was recorded on the patient monitor as an event, which we recalled and printed (Fig. 2).

This case report was approved by the Institutional Review Board (IRB) of St. Mary’s Hospital, Catholic University of Korea (approval number SC22ZISE0053). The patient provided written informed consent for the publication of this report.

## 3. Discussion

Cardiac arrest in the perioperative period can occur for several reasons. Common causes include hypoxia, hypovolemia, and increased vagal activity due to medications routinely used during general anesthesia or surgical stimulation.^[5]^ The incidence of trigeminal reflex in maxillofacial surgical procedures is approximately 1.6%.^[6]^ It occurs in a wide variety of surgeries, including craniofacial surgery, craniofacial pain surgery, skull base neurosurgery, maxillofacial surgery, mechanical stimulation of ocular and periocular structures, rhinoplasty, and blepharoplasty.^[7]^ However, the occurrence of reflexes in BDET has not been reported. BDET as a treatment for ETD has success rates ranging from 64% to 97%, and a complication rate of approximately 2%, suggesting a good safety profile.^[2]^ As complications, however, minor tears of the mucosal lumen, epistaxis, the exacerbation of tinnitus, temporary deafness or vertigo, as well as subcutaneous emphysema and patulous ETD have been reported after BDET.^[4]^ To our knowledge, this case of transient asystole is the first significant general complication of BDET.

The afferent limb of the trigeminal reflex is the three branches of the trigeminal nerve, the Gasserian ganglion, or the intracranial part of the trigeminal nerve. The maxillary nerve, a branch of the trigeminal nerve, carries sensory information from the nares and upper lip, upper teeth and gums, nasal mucosa, palate, roof of the pharynx, and vicinity of the ET orifice. The insertion of endoscope or balloon into the nasopharynx may have caused the trigeminocardiac reflex, inducing asystole in this case. However, the cardiac rhythm on EKG not change during the procedure. When we considered the plausibility and reversibility of the cause‐effect relationship of the reflex,^[8]^ the appearance of asystole was apparently related to balloon inflation because asystole appeared promptly after inflation, and deflation abolished the asystole. The reflex did not seem to be related to the insertion of the endoscope or balloon into the nasopharynx.

The innervation of the mucous membrane lining the ET is primarily from the tympanic plexus, which receives its major contribution from the tympanic nerve, a branch of the glossopharyngeal nerve.^[9]^ If balloon inflation provokes the vagal reflex, the afferent limb should be the glossopharyngeal nerve. Although there was no declared glossopharyngeal-vagal reflex, the occurrence of vagal syncope during glossopharyngeal neuralgia indicates that there should be a relationship between the glossopharyngeal and vagus nerves.^[10]^ Most proposed mechanisms are based on the close anatomical relationship between the glossopharyngeal and vagus nerves in the medulla oblongata and the possibility of vagoglossopharyngeal reflex arc formation.^[11]^ Another view suggests that some visceral sensory fibers of the glossopharyngeal nerve might connect with fibers of the carotid sinus nerve (nerve of Hering), stimulating the latter; therefore, activation of the baroreceptors of the carotid sinus, resulting in bradycardia, asystole, premature atrial contractions, or hypotension that can lead to syncope.^[12]^

Stimulation of the intracranial rootlets of the glossopharyngeal and vagus nerves can also provoke the trigeminocardiac reflex through their projections into the spinal trigeminal tract but before their connection to the nucleus tractus solitarii. It seems that the spinal trigeminal system serves as a site of integration of depressor reflexes from receptors in the head and neck.^[13]^ The asystole in this case could be a trigeminocardiac reflex provoked by the glossopharyngeal nerve that projected into the spinal trigeminal tract. It is important to note that BDET itself can induce the vagal reflex.

During BDET, the change in middle ear pressure depends on the speed of inflation and maximum inflation pressure.^[14]^ Slow dilation at 1 atm per second up to 12 atm is recommended. Slow dilation minimizes the risk of triggering an isobarometric arc and makes the procedure more comfortable for the patient.^[4]^ But, it is not certain whether the speed of balloon inflation is related to the vagal reflex.

BDET is known that BDET is effective in reducing ETD symptoms in patients in office and operating room settings. However, BDET, which is known to be safe, is associated with the occurrence of a neutrally mediated vagal reflex. Our experience with transient asystole by balloon inflation during BDET alerts anesthesiologists to pay proper attention to monitoring and potential danger.

## Author contributions

**Conceptualization:** MinJung Shin, SeungHee Cha.

**Visualization:** MinJung Shin, SeungHee Cha.

**Writing – original draft:** MeeYoung Chung.

**Writing – review & editing:** JiYung Lee.
